# Ciclopirox Olamine Exerts Tumor-Suppressor Effects *via* Topoisomerase II Alpha in Lung Adenocarcinoma

**DOI:** 10.3389/fonc.2022.791916

**Published:** 2022-02-18

**Authors:** Jie Yin, Gang Che, Kan Jiang, Ziyang Zhou, Lingyun Wu, Mengyou Xu, Jian Liu, Senxiang Yan

**Affiliations:** ^1^ Department of Radiation Oncology, The First Affiliated Hospital, Zhejiang University School of Medicine, Hangzhou, China; ^2^ Department of Surgical Oncology, The First Affiliated Hospital, Zhejiang University School of Medicine, Hangzhou, China; ^3^ Department of Medical Oncology, Peking University Cancer Hospital, Beijing, China

**Keywords:** lung adenocarcinoma, ciclopirox olamine, topoisomerase II alpha, DNA damage, apoptosis

## Abstract

**Background:**

Globally, lung cancer is one of the most malignant tumors, of which lung adenocarcinoma (LUAD) is the most common subtype, with a particularly poor prognosis. Ciclopirox olamine (CPX) is an antifungal drug and was recently identified as a potential antitumor agent. However, how CPX and its mechanism of action function during LUAD remain unclear.

**Methods:**

The effects of CPX on cell proliferation, cell cycle, reactive oxygen species (ROS) levels, and apoptosis were assessed using the 3-(4,5-dimethylthiazol-2-yl)-2,5-diphenyl-2H-tetrazolium bromide (MTT) assay, colony formation, western blotting, flow cytometry assays, and immunohistochemistry. Global gene expression levels were compared between control and CPX-treated LUAD cells. A LUAD xenograft mouse model was used to evaluate the potential *in vivo* effects of CPX.

**Results:**

We observed that CPX displayed strong antitumorigenic properties in LUAD cells, inhibited LUAD proliferation, induced ROS production, caused DNA damage, and activated the ATR-CHK1-P53 pathway. Topoisomerase II alpha (TOP2A) is overexpressed in LUAD and associated with a poor prognosis. By analyzing differentially expressed genes (DEGs), TOP2A was significantly down-regulated in CPX-treated LUAD cells. Furthermore, CPX treatment substantially inhibited *in vivo* LUAD xenograft growth without toxicity or side effects to the hematological system and internal organs.

**Conclusions:**

Collectively, for the first time, we showed that CPX exerted tumor-suppressor effects in LUAD *via* TOP2A, suggesting CPX could potentially function as a promising chemotherapeutic for LUAD treatment.

## Introduction

Lung cancer is one of the most malignant tumors with a poor prognosis, and is one of the leading causes of cancer death worldwide (1). Lung adenocarcinoma (LUAD) is the most common lung cancer subtype, with a particularly poor prognosis (2). Most patients with LUAD are diagnosed too late for effective surgical treatment (3). Despite advances in multi-modality therapies, such as gene-targeted therapy and immunotherapy which have improved the prognosis of patients with LUAD, the mortality rate of LUAD patients remains dismal (4–6). Chemotherapy is the main treatment strategy for advanced LUAD (7), however, a lack of prognostic biomarkers and unsatisfactory curative effects limit survival outcomes for LUAD patients (8). Therefore, identifying novel LUAD treatments is vital.

Ciclopirox olamine (CPX) is a synthetic antifungal agent with several decades of clinical application; it demonstrates broad-spectrum activity and inhibits dermatophytes, yeasts, molds, and many Gram-positive and Gram-negative pathogens (9). However, CPX was recently reported to have potential antitumor effects. Initially, CPX was reported as a reversible cell cycle inhibitor with tumor suppressor effects in hematological malignancies (10). Further research showed CPX bound and chelated intracellular iron which was functionally important for its cytotoxicity effects in leukemia and myeloma (11, 12). Subsequently, CPX inhibited myeloma by targeting the Wnt/β-catenin pathway, both *in vivo* and *in vitro* (13–15). Apart from Wnt (16), β-catenin, and c-Myc signaling (17), CPX exerted anti-leukemic effects by specifically inhibiting mammalian target of rapamycin (mTOR) (18). Critically, in a phase I study in patients with advanced hematological malignancies, oral CPX was safe with tolerable doses (19). Furthermore, CPX inhibited pancreatic cancer (20), and also cervical cancer cell proliferation by targeting eIF5A (21).

CPX also showed antitumor activities against several cancers, including inhibition of breast cancer by mediating apoptosis through a caspase-dependent pathway (22, 23), driving colorectal cancer cell death by activating PERK-dependent endoplasmic reticulum stress and targeting DJ-1 (24–26), having a therapeutic role in head and neck squamous cell carcinoma as an iron chelator (27), targeting histone demethylases in MYC-driven neuroblastomas (28), inhibiting mTORC1 signaling by activation of AMPK and activating ATR-Chk1 signaling pathway leading to Cdc25A protein degradation in rhabdomyosarcoma (29, 30). Recently, a study comparing tumor-related signaling pathways with known compounds reported that CPX had potential roles in LUAD treatment (31). However, the mechanism of CPX-mediated tumor suppression remains unclear, thus limiting and hindering its use in preclinical and clinical studies. In this study, we will address this knowledge gap by investigating the role of CPX in LUAD. In this study, we found that CPX may inhibit LUAD *via* topoisomerase II alpha (TOP2A).

The DNA topoisomerase family has two subfamilies: type I DNA topoisomerases cleave single-stranded DNA, whereas type II topoisomerases break double-stranded DNA (32) and resolve sophisticated DNA topological intermediates generated during DNA recombination, replication, transcription, and repair processes, such as relaxed, supercoiled, knotted, and catenated DNA (33). The human genome has two types II topoisomerases: TOP2A and topoisomerase IIβ (34). Topoisomerase IIβ is not essential for cell survival and proliferation (35), whereas TOP2A is a cell proliferation biomarker and is overexpressed in several rapidly proliferating cancers (36–41), including LUAD (42, 43). Additionally, TOP2A, as a prognostic factor and oncogene, is associated with LUAD survival, breast cancer, and prostate cancer (42–45). It was reported that TOP2A induced malignant characteristics in pancreatic cancer by activating β-catenin signaling (40). TOP2A knockdown reduced extracellular regulated protein kinases (ERK) and serine/threonine kinase Akt phosphorylation levels in colon cancer (46). Furthermore, TOP2A was identified as a therapeutic target for breast cancer, small-cell lung cancer, ovarian cancer, and leukemia (47–49). Previous studies showed that TOP2A functioned as an oncogene, played critical roles in LUAD cells, was associated with malignant progression, and predicted poor prognosis in LUAD patients (42, 43). Moreover, the TOP2A targeting of CCNB1 and CCNB2 promoted LUAD cell proliferation and metastasis (43) and activated ERK/JNK/p-P38/CHOP signaling in LUAD (42).

Recent research has suggested TOP2A is a core gene in the LUAD-signaling network (50). However, the biological function of TOP2A in LUAD remains unclear and few TOP2A inhibitors are available for LUAD. Therefore, TOP2A is a major candidate gene worthy of further research in LUAD.

Similarly, CPX function in LUAD remains unclear, while underlying CPX inhibitory mechanisms, *via* TOP2A signaling, are unreported. To address this, we investigated CPX-mediated suppression of LUAD cell growth both *in vitro* and *in vivo.* Our data suggested that down-regulating TOP2A was associated with CPX anti-tumor activity. Furthermore, our preliminary evidence suggests CPX could be repurposed as an anticancer agent for LUAD.

## Methods

### Cell Culture

The human non-small cell lung cancer (NSCLC) cell lines, A549, PC9, and H1299 were obtained from the Cell Bank of Shanghai, China. Cells were cultured in Dulbecco’s Modified Eagle Medium (DMEM) supplemented with 10% fetal bovine serum (BI), 100 U/mL penicillin (Sigma), and 10 mg/L streptomycins (Sigma) in a humidified incubator at 37°C in 5% CO_2_. Cells were routinely passaged using standard cell culture techniques.

### The 5-Ethynyl-2´-deoxyuridine Assay

Cell proliferation was determined using the EdU assay. Cells grown on coverslips were treated with CPX for 24 h and stained with EdU (RiBoBio) for 12 h. Coverslips were processed according to the manufacturer’s instructions. Finally, nuclei were stained with 4´,6-diamidino-2-phenylindole (DAPI) for 10 min, and cells were observed using fluorescence microscopy (Olympus).

### Reagents and Antibodies

The following reagents and primary antibodies were used: CPX (MedChemExpress), N-Acetyl-cysteine (MedChemExpress), GAPDH (Santa Cruz Biotechnology), Actin (Proteintech, 20536-1-AP), PARP (Cell Signaling Technology, 9532), Cleaved PARP (Cell Signaling Technology, 5625), Cleaved caspase-3 (Cell Signaling Technology, 9664), CDK4 (Santa Cruz Biotechnology, sc-23896), CDK6 (Santa Cruz Biotechnology, sc-7961), Rb (Santa Cruz Biotechnology, sc-102), cyclin D_3_ (Cell Signaling Technology, 2936), DJ-1 (Abcam, ab76008), phosphorylated-P53 (p-P53) (Cell Signaling Technology, 9286), p-ATR (Cell Signaling Technology, 2853), p-Chk1 (Cell Signaling Technology, 2348), p-H_2_AX (Millipore, 05-636), Ki-67 (Abcam, ab16667), and TOP2A (Abcam, ab52934).

### Cytotoxicity Assay

Cell viability was determined using the Invitrogen Countess Automated Cell Counter (ThermoFisher) and the 3-(4,5-dimethylthiazol-2-yl)-2,5-diphenyl-2H-tetrazolium bromide assay (MTT). Cell counting was conducted using trypan blue. For the MTT assay, cells were seeded overnight in 96-well plates (Corning) for treatment. Then, to each well, 20 μL of 5 mg/mL MTT solution was added (Sigma), after which, 150 μL dimethyl sulfoxide was added to dissolve formazan. The optical density was read at 490 nm using a microplate reader (Bio-Rad). Cell viability (%) = Absorbance at 490 nm (treated-blank)/A490 (control-blank) × 100%.

### Colony Formation Assay

Exponentially growing cells were incubated with or without CPX for 12–14 days at 37°C in 5% CO_2_. Colonies were fixed in 70% ethanol and stained in 0.5% crystal violet (Sigma) for 30 min. Colonies comprised ≥ 50 cells and were visible to the naked eye.

### Apoptosis Assay

Cells were plated in 6-well plates overnight and treated with or without CPX. After 24 h, cells were harvested and stained in Annexin V-PE/7-AAD (BD) for 15 min, followed by apoptosis analysis by flow cytometry (BD FACS Calibur).

### Cell Cycle Assay

Cells were plated in 6-well plates overnight and treated with or without CPX. After 24 h, cells were fixed in 75% ethanol, incubated with RNase and PI for 30 min. Cell cycle analysis was determined by flow cytometry (BD FACS Calibur).

### Reactive Oxygen Species Assay

Cells were seeded in 6-well plates at 4×10^5^ in DMEM and cultured overnight at 37°C in 5% CO_2_. The medium was then replaced with a medium containing a gradient concentration of CPX. After 24 h, fluorescence microscopy (Olympus) was used to capture images. Cells were also harvested and intracellular ROS levels were determined using a 2′,7′-Dichlorofluorescin diacetate (DCFH-DA) fluorescent probe according to the manufacturer’s instructions. Stained cells were analyzed by flow cytometry (BD FACS Calibur).

### Western Blotting

Proteins were extracted in RIPA buffer and concentrations were determined using the Pierce BCA assay (ThermoFisher). Proteins were resolved using sodium dodecyl sulfate-polyacrylamide gel electrophoresis and transferred to nitrocellulose or polyvinylidene fluoride membranes (Whatman). After blocking in 5% defatted milk, membranes were incubated overnight at 4°C with primary antibodies. Then, after washing, membranes were incubated with a horseradish peroxidase-conjugated secondary antibody at room temperature for 1 h. Protein signals were detected using a SuperSignal West FemtoMaximum Sensitivity Substrate, and membranes imaged using a ChemiScopeTouch series fluorescence and chemiluminescence imaging system (CLINX).

### Immunofluorescence

Cells grown on coverslips were fixed in 4% paraformaldehyde, permeabilized in 0.2% triton, and blocked in 1% bovine serum albumin and 0.5% goat serum in phosphate-buffered saline (PBS). Then, cells were stained with γ-H_2_AX overnight. Nuclei were stained with DAPI (Sigma) for 5 min. Fluorescence microscopy (Olympus) was used to visualize cells.

### RNA Sequencing

Total RNA was isolated and purified using TRIzol reagent (Invitrogen, Carlsbad, CA, USA) following the manufacturer’s instructions. RNA quantity and purity were assessed using a NanoDrop ND-1000 (NanoDrop, Wilmington, DE, USA). RNA integrity was assessed using an Agilent 2100 with RNA integrity (RIN) number > 7.0. Poly(A) RNA was purified from total RNA (5 μg) using poly-T oligo-attached magnetic beads over two purification rounds. Purified poly(A) RNA was then fragmented using divalent cations at a high temperature. Then, cleaved fragments were reverse-transcribed to create cDNA, which was used to synthesize U-labeled second-strand DNA using *Escherichia coli* DNA polymerase I, RNase H, and dUTP. Then, an A-base was added to the blunt end of each strand for ligation to indexed adapters. Each adapter contained a T-base overhang for adaptor ligation to the A-tailed fragmented DNA. Single- or dual-index adapters were ligated to fragments, and size selection was performed using AMPureXP beads. After the heat-labile UDG enzyme treatment of the U-labeled second-stranded DNAs, the ligated products are amplified with PCR by the following conditions: initial denaturation at 95°C for 3 min; 8 cycles of denaturation at 98°C for 15 sec, annealing at 60°C for 15 sec, and extension at 72°C for 30 sec; and then final extension at 72°C for 5 min. The average insert size for the final cDNA library was 300 bp ( ± 50 bp). At last, we performed the 150bp paired-end sequencing on an Illumina Hiseq 4000 (LC Bio, China) following the vendor’s recommended protocol.

### Transfection

Transfections with small interfering RNAs (siRNAs) were performed using GP-transfect-Mate Transfection Reagent (GenePharma) according to the manufacturer’s instructions. siRNAs were purchased from GenePharma. Details can be found in Supplemental Methods and Materials.

### The Xenograft Tumor Model

Six-week-old Balb/c male nude mice were obtained from the Zhejiang Academy of Medical Sciences (Hangzhou, China). Animals were housed under specific pathogen-free conditions. In total, 1× 10^7^ A549 and PC9 cells were suspended in PBS and injected subcutaneously into the right flank of each mouse to generate xenograft tumors. When the tumor volume reached an average of 80–100 mm^3^, mice were randomly divided into two groups of six; physiological saline (0.9% NaCl) group and a CPX-treated group.

Mice received intraperitoneal injections of CPX (25 mg/kg) dissolved in 0.9% NaCl or 0.9% NaCl alone (saline control) once a day for 15 days. Tumor volumes were evaluated at indicated time points using the formula: volume = 1/2 ×length×width^2^. Mice were weighed every 3 days. After 15 days, mice were humanely sacrificed, photographed, and tumors dissected, weighed, fixed, and embedded. Heart, liver, spleen, lung, kidney, and tumor specimens were isolated and immediately fixed in 10% formalin. All animal experiments were carried out following Institutional Animal Care and Use Committee guidelines, University Laboratory Animal Research of Zhejiang University. The experiments were conducted as per protocol approved by the local hospital ethics committee.

### Immunohistochemical Staining

For histopathological and IHC analyses, heart, liver, spleen, lung, kidney, and tumor tissue specimens were fixed in 10% neutral buffered formalin, embedded in paraffin, sectioned (5 μm), and stained in hematoxylin and eosin (H&E). Sections were randomly numbered before observation by an experienced pathologist. Ki-67, p-H_2_AX, and TOP2A expression levels were evaluated using specific antibodies. Apoptotic cells were detected by TUNEL staining according to the manufacturer’s instructions. Images were acquired using the Panoramic 250 FLASH scanner (3DHISTECH) equipped with scanner software.

### TOP2A Validation Using The Cancer Genome Atlas

UALCAN (http://ualcan.path.uab.edu/) is an online service that provides easy access to publicly available cancer OMICS data (e.g., TCGA, MET500, and CPTAC). A boxplot was used to compare TOP2A mRNA expression levels in LUAD tissue when compared with paired normal lung tissue. To validate TOP2A expression levels correlations with patient survival and LUAD pathological stages, Kaplan-Meier survival curves were generated. TOP2A mRNA expression levels above or below the median were used to classify patients into high and low expression groups.

### Statistical Analysis

Statistical analyses were performed in Prism 8 (GraphPad Software, San Diego, CA, USA). Data were expressed as the mean ± standard deviation. Statistical comparisons between groups were made using the Student’s t-test. P < 0.05 was defined as statistically significant. *P < 0.05; **P < 0.01; ***P < 0.001; ****P < 0.0001, and ns = non-significant.

## Results

### CPX Inhibits *In Vitro* LUAD Cell Proliferation

**Figure 1:**
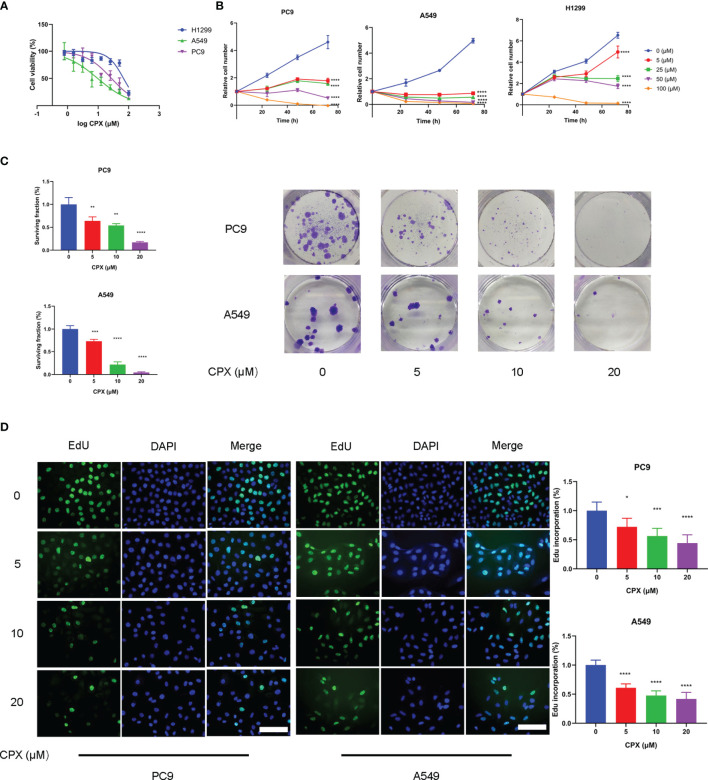
CPX inhibits LUAD cell proliferation *in vitro*. **(A)** A549, PC9 and H1299 cells were treated with indicated CPX concentrations for 24 hours, and cell viability was determined by MTT assay. **(B)** LUAD cells were treated with CPX at the indicated concentration and cell growth was assessed at 24 h, 48 h, and 72 h by MTT assay. **(C)** Colony formation assay in PC9 and A549 cells treated with indicated CPX concentrations for 24 hours. Cells were seeded in 6-well plates for two weeks and colony numbers quantified. **(D)** EdU assay of LUAD cells treated with indicated CPX concentrations for 24 hours and EdU incorporation quantitated. Scale bar = 100 μm. *P < 0.05; **P < 0.01; ***P < 0.001; ****P < 0.0001. Statistical significance compared with respective control groups.

CPX antitumor effects were investigated using LUAD cell line responses (A549, PC9, and H1299) to CPX. Briefly, cells were treated with different CPX concentrations for 24 h and cell viability was assessed using the MTT assay (**Figure 1A**). To determine cell proliferation curves, we treated cells with indicated CPX concentrations and cell numbers measured at different time points (24 h, 48 h, and 72 h) by MTT assay (**Figure 1B**). CPX significantly suppressed cell viability and proliferation in a dose and time-dependent manner (**Figures 1A, B**). CPX half inhibitory concentrations in A549, PC9 and H1299 cells were 4.4 μM, 27.4 μM, and 67.9 μM respectively; therefore, A549 and PC9 cells were more CPX-sensitive and were used in subsequent experiments. Reduced colony formation levels and EdU incorporation also indicated CPX inhibitory effects on cell proliferation in a dose-dependent manner (**Figures 1C, D**).

### CPX Induces Cell Cycle Arrest and Apoptosis

**Figure 2:**
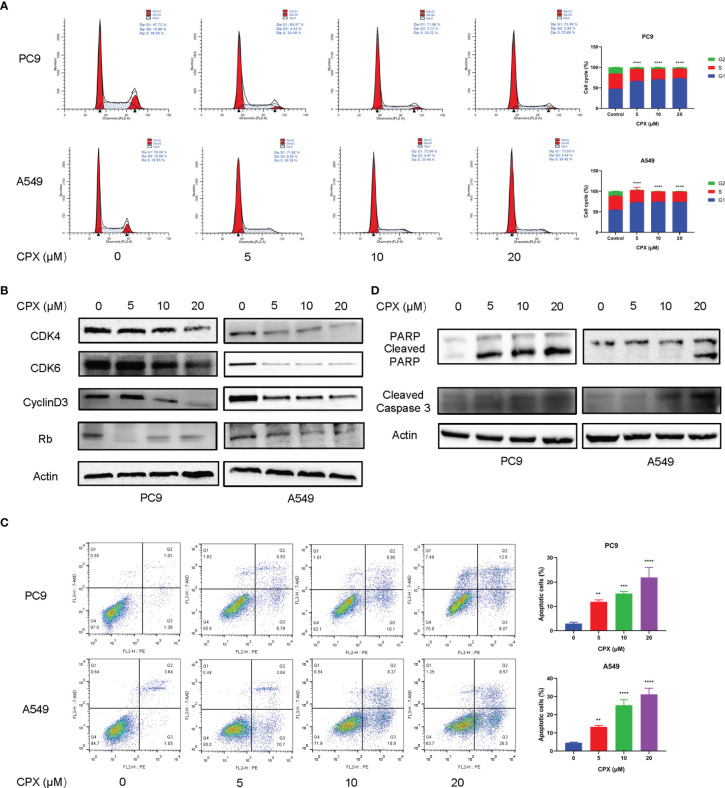
CPX induces cell cycle arrest and apoptosis. **(A)** Cell-cycle distribution was analyzed by flow cytometry after cells were treated with indicated CPX concentrations for 24 hours. **(B)** Western blotting of cell cycle-related protein levels in cells treated with indicated CPX concentrations for 24 hours. **(C)** Cells treated with indicated CPX concentrations for 24 hours were fixed, stained with PE/7-AAD, and analyzed by flow cytometry. **(D)** Western blotting of apoptosis-related proteins in cells treated with indicated CPX concentrations for 24 hours. **P < 0.01; ***P < 0.001; ****P < 0.0001. Statistical significance compared with respective control groups.

To investigate CPX anticancer mechanisms in LUAD, we examined if CPX affected cell cycle distributions and observed G1-phase arrest (**Figure 2A**). Moreover, we also examined the expression of cell cycle-related proteins in CPX-treated LUAD cells. As expected, CPX treatment significantly reduced cell cycle-related protein levels, including cyclin D_3_, Rb, CDK4, and CDK6 (**Figure 2B**). Apoptosis was also observed by flow cytometry (**Figure 2C**). In addition, CPX treatment increased PARP, cleaved PARP, and cleaved caspase 3 (specific and sensitive apoptosis markers) levels in LUAD cells (**Figure 2D**). Thus, CPX displayed considerable antitumor effects in LUAD cells *in vitro* by arresting the cell cycle and inducing apoptosis.

### CPX Increases Intracellular ROS Levels

**Figure 3:**
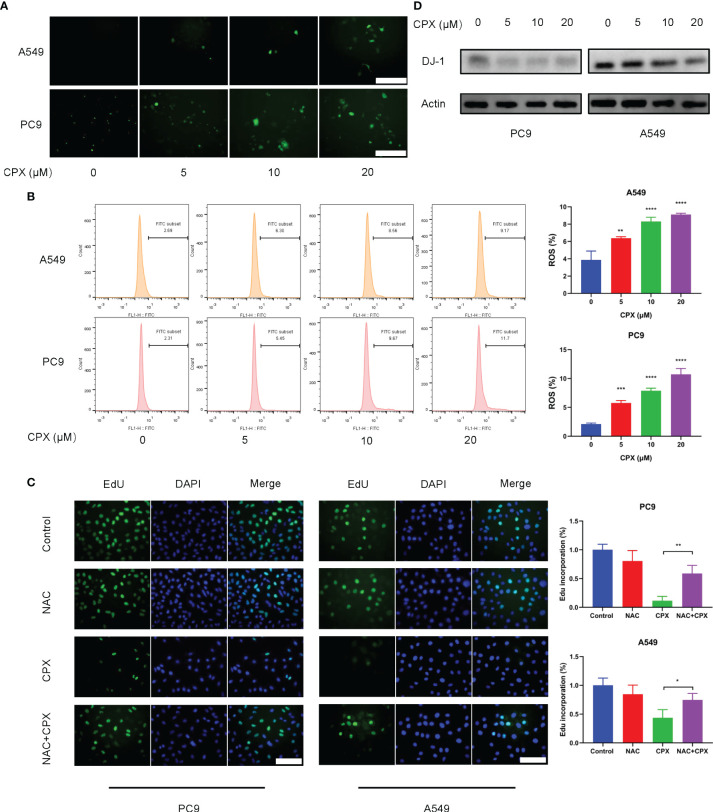
CPX increases intracellular ROS levels. **(A)** Cells treated with indicated CPX concentrations were stained with DCFH-DA and photographed under fluorescence microscopy to evaluate ROS levels. Scale bar = 100 μm. **(B)** Flow cytometry was used to analyze ROS levels in cells treated with indicated CPX concentrations by DCFH-DA staining. **(C)** EdU assay showing LUAD cells treated with or without 2 mM NAC in the presence or absence of 20 μM CPX for 24 hours. Scale bar = 100 μm. **(D)** Western blot showing DJ-1 levels in LUAD cells pretreated with indicated CPX concentrations for 24 hours. *P < 0.05; **P < 0.01; ***P < 0.001; ****P < 0.0001. Statistical significance compared with respective control groups.

Previous studies suggested that CPX exerted its antitumor activities by inducing intracellular ROS levels (25). To see if ROS was involved in CPX-induced cytotoxicity in LUAD cells, IF was used to detect ROS expression levels in cells treated with different CPX concentrations. Cellular ROS levels were positively correlated with CPX concentrations (**Figure 3A**), as confirmed by flow cytometry (**Figure 3B**). Also, western blotting showed that expression of the anti-oxidative stress molecule, DJ-1 was decreased (**Figure 3D**). Next, we investigated the role of ROS in CPX-induced growth suppression. We observed that the combined application of CPX with the ROS scavenger, N-Acetyl-cysteine (NAC) restored CPX-induced cell growth suppression, as evidenced by EdU incorporation in combinatorial treatments (**Figure 3C**).

### CPX Induces DNA Damage

**Figure 4:**
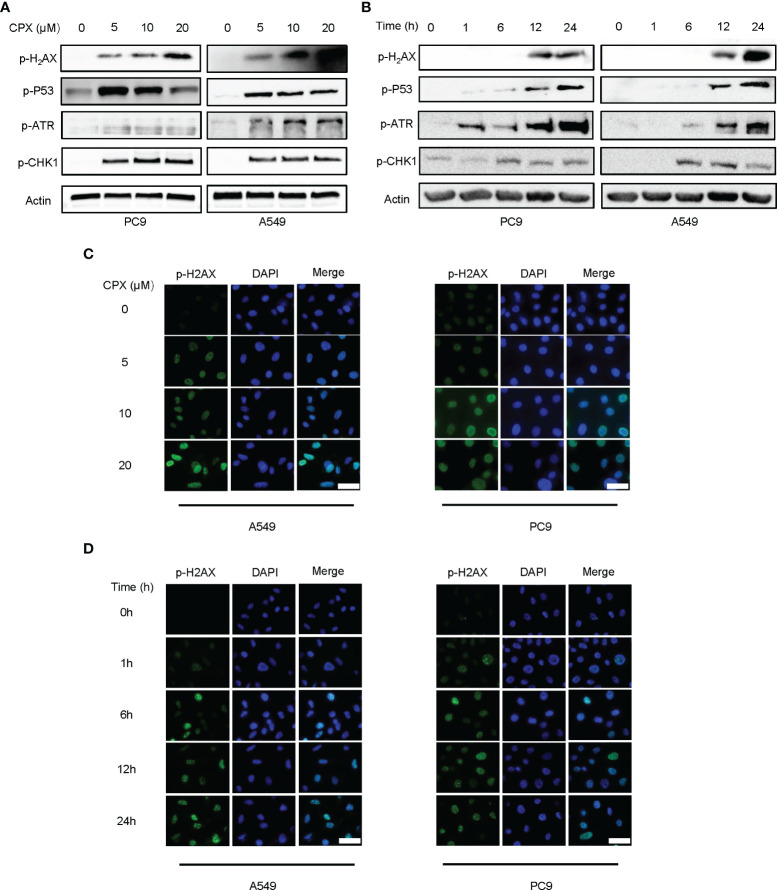
CPX induces DNA damage and repair. **(A)** Western blotting showed p-H_2_AX p-ATR, p-CHK1, and p-P53 levels in LUAD cell lines pretreated with indicated CPX concentrations for 24 hours. **(B)** Western blotting showed p-H_2_AX, p-ATR, p-CHK1, and p-P53 levels at different time points after treatment with 5 μM CPX. **(C)** Immunofluorescence images showing p-H_2_AX staining in LUAD cells pretreated with indicated CPX concentrations for 24 hours. **(D)** Immunofluorescence images showing p-H_2_AX staining at different time points after treatment with 5 μM CPX. Scale bar = 100 μm.

Since DNA damage and repair are features of ROS-induced cell effects (51), we examined if CPX was involved in DNA damage responses and subsequent DNA repair. Phosphorylated H_2_AX (p-H_2_AX) is a DNA double-strand break (DSB) biomarker. Western blotting and IF data showed CPX treatments induced DNA damage in LUAD cells in a dose and time-dependent manner (**Figures 4A–D**). As ATR-CHK1 and P53 pathways have essential roles in DNA damage responses (DDRs), we assessed the ATR-CHK1 pathway and P53 phosphorylation activities after CPX treatment. As shown (**Figures 4A, B**), CPX activated phosphorylated ATR-CHK1 and phosphorylated P53 in LUAD cells. Together, CPX induced cellular ROS accumulation and DNA damage.

### CPX Inhibits *In Vivo* Tumor Growth

**Figure 5:**
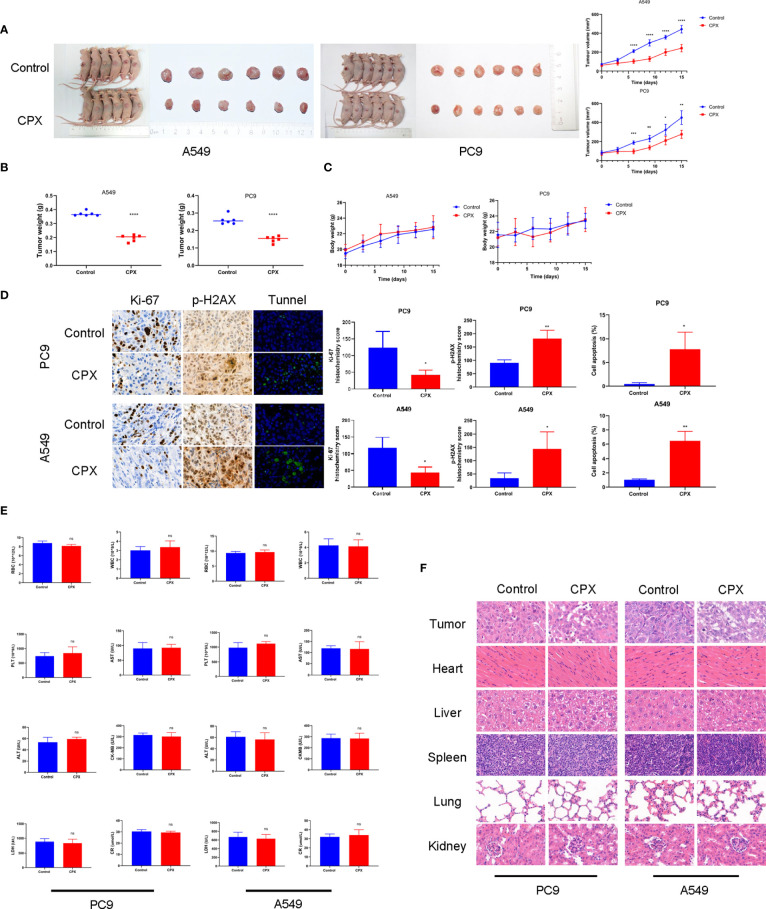
CPX inhibits *in vivo* tumor growth without side effects. **(A)** Dissected tumor images from A549 and PC9 xenograft models. Tumor volumes were evaluated at indicated time points. **(B)** Tumor weights. **(C)** Bodyweight growth curves in xenograft models. **(D)** Immunohistochemistry showing Ki67and p-H_2_AX staining, and Tunnel assay of xenograft tumors (magnification 400×). **(E)** Red blood cell (RBC), white blood cell (WBC), platelet (PLT) counts, aspartate aminotransferase (AST), alanine aminotransferase (ALT), creatine kinase isoenzyme (CK-MB), lactate dehydrogenase (LDH), and creatinine (CR) levels. **(F)** Representative hematoxylin & eosin images of mouse tumors and organs (magnification 400×). *P < 0.05; **P < 0.01; ***P < 0.001; ****P < 0.0001, ns, non-significant. Statistical significance compared with respective control groups.

We next evaluated the therapeutic effects of CPX-induced tumor suppression *in vivo*. As shown (**Figure 5A**), CPX significantly inhibited LUAD xenograft growth when compared with controls. Similarly, tumor weight was also reduced in the CPX-treated group (**Figure 5B**). CPX treatment was well-tolerated with no notable body weight loss (**Figure 5C**). IHC was consistent with previous results; CPX decreased Ki-67 staining (a cell proliferation marker) and increased p-H_2_AX and Tunnel (apoptosis marker) staining in tumor tissue (**Figure 5D**).

We also investigated if CPX caused additional side effects in the hematological system and other organs. As shown (**Figure 5E**), no significant differences were observed in red blood cell (RBC), white blood cell (WBC), and platelet (PLT) counts. Furthermore, no abnormalities were observed in other liver indicators, including aspartate aminotransferase (AST), alanine aminotransferase (ALT), creatine kinase isoenzymes (CK-MB), lactate dehydrogenase (LDH), and creatinine (CR). Similarly, no notable morphological changes were observed in organ tissues (**Figure 5F**). Together, CPX displayed antitumor effects and was safely administered *in vivo*.

### CPX Down-Regulates TOP2A in LUAD

**Figure 6:**
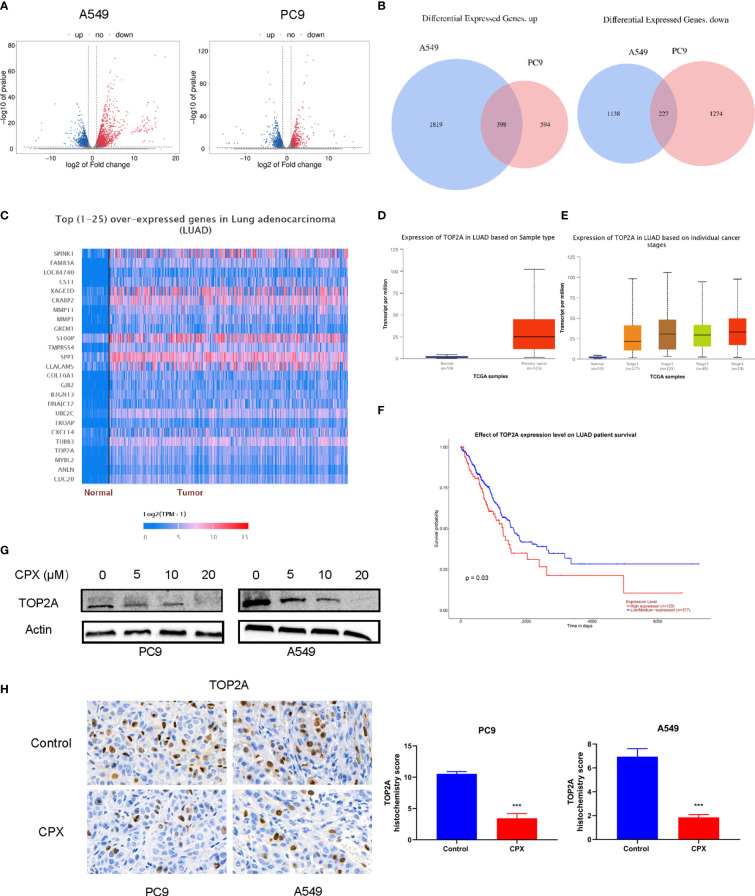
CPX down-regulates TOP2A in LUAD. **(A)** Volcano plot analysis of DEGs in A549 and PC9 cells treated with or without 20 μM CPX for 24 hours. Red areas indicate significant overexpression, green areas indicate significant down-regulation, and grey areas indicate non-significant changes. **(B)** Venn diagram intersections show up-or down-regulated DEGs in A549 and PC9 cells. **(C)** Heatmap of the top (25) over-expressed genes in LUAD (UALCAN). **(D)** A box plot showing TOP2A expression in LUAD (UALCAN). **(E)** Box plot showing TOP2A expression at different tumor stages among LUAD patients (UALCAN). **(F)** Kaplan-Meier plot showing the effects of TOP2A expression levels on LUAD patient survival (UALCAN). **(G)**. Western blotting showing TOP2A expression in LUAD cells pretreated with indicated CPX concentrations for 24 hours. **(H)**. TOP2A immunohistochemistry staining in xenograft tumors in different groups (magnification 400×). ***P < 0.001. Statistical significance compared with respective control groups.

To identify molecular mechanisms underpinning CPX-induced LUAD suppression, we investigated global gene expression patterns in CPX-treated cells and compared them with controls using RNA-seq. Using a ≥ 2-fold change (FC) and < 0.05 P-value as a cut-off to define overexpression or down-regulation, we identified a series of differentially expressed genes (DEGs) between CPX-treated and control groups (**Figure 6A**). Next, using a Venn diagram, we identified 398 co-upregulated and 227 co-down-regulated genes in A549 and PC9 cells (**Figure 6B**). Then, we screened down-regulated oncogenes from DEGs, compared their prognostic differences, and reviewed related studies. Among these DEGs, TOP2A was identified and was previously shown to impact LUAD occurrence, development, and prognosis (42, 43). TOP2A expression was down-regulated and it was one of the top 25 highly expressed genes in LUAD (**Figure 6C**). Furthermore, UALCAN was used to validate TOP2A differential mRNA expression analysis in tumor and normal tissues, and showed TOP2A was overexpressed in LUAD (**Figure 6D**). Thus, TOP2A mRNA overexpression was strongly associated with LUAD occurrence and development (**Figure 6E**). Patients with high TOP2A mRNA expression levels had lower overall survival (OS) than patients with low expression (**Figure 6F**). Given these data, we performed western blotting to determine TOP2A expression in cells after CPX treatment; expression was significantly decreased in CPX-treated LUAD cells (**Figure 6G**). IHC also showed that TOP2A expression in the CPX group was significantly reduced in animal xenograft tumor samples (**Figure 6H**). Therefore, TOP2A may be a therapeutic target for LUAD treatment.

### TOP2A Knockdown Enhances the Anti-Tumor Effects of CPX

**Figure 7:**
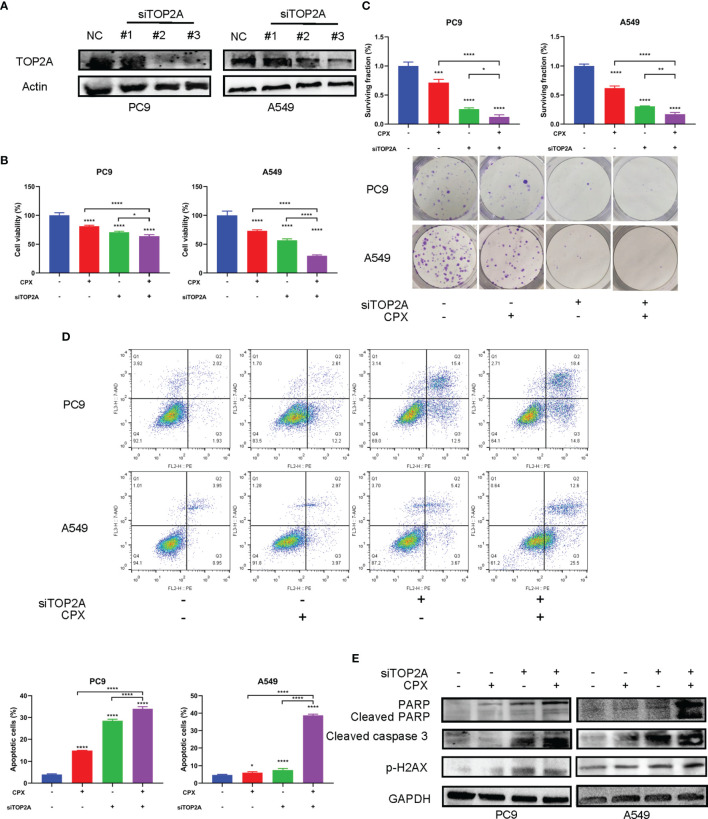
TOP2A knockdown enhances CPX anti-tumor effects. **(A)** Western blot showing TOP2A expression at 48 hours post siRNA transfection. **(B)** Cells were transfected with TOP2A siRNA, 5 μM CPX, and combinations thereof; 24 hours later, cell viability was assessed using the MTT assay. **(C)** Cells were transfected with TOP2A siRNA, 5 μM CPX, and combinations thereof; 24 hours later, cells were seeded into 6-well plates for colony formation assays. **(D)** Cells treated with TOP2A siRNA, 5 μM CPX, and combinations thereof for 24 hours, fixed, stained with PE/7-AAD, and analyzed by flow cytometry. **(E)** Western blotting showing PARP, cleaved PARP, cleaved caspase3, and p-H_2_AX expression in LUAD cells pretreated with TOP2A siRNA, 5 μM CPX, and the combination thereof for 48 hours. *P < 0.05; **P < 0.01; ****P < 0.0001. Statistical significance compared with respective control groups.

We speculated that the anti-tumor effects of CPX in LUAD could be associated with TOP2A regulation. Therefore, we investigated if TOP2A knockdown enhanced the anti-tumor effects of CPX in LUAD cells. TOP2A was knocked down using siRNAs, after which western blotting was performed to verify knockdown (**Figure 7A**). MTT and colony formation assays showed that knockdown significantly enhanced CPX anti-proliferation effects (**Figures 7B, C**). Additionally, knockdown increased apoptosis (**Figure 7D**). Also, knockdown induced DNA damage; cleaved PARP and p-H_2_AX expression levels were both increased (**Figure 7E**). Additionally, these effects were enhanced after TOP2A knockdown was combined with CPX in cells (**Figures 7B–E**). Thus, CPX down-regulated TOP2A expression and TOP2A knockdown further enhanced CPX anti-tumor effects, suggesting that at least part of the CPX effects on LUAD were mediated by TOP2A.

## Discussion

For the first time, we showed that CPX possessed anti-LUAD cancer effects both *in vitro* and *in vivo*. CPX inhibited cell proliferation, promoted ROS accumulation, DNA damage, cycle arrest, and apoptosis. Moreover, CPX showed anti-tumor effects in a xenograft model, with no toxicity to the peripheral blood system and organs. CPX significantly down-regulated TOP2A expression, and TOP2A knockdown combined with CPX exerted synergistic anti-tumor effects. Therefore, CPX mechanisms during LUAD appear to be closely associated with TOP2A expression.

CPX is an off-patent antifungal agent and possesses anticancer effects toward various cancers by inhibiting cell growth, inducing cell death, and suppressing angiogenesis (11, 22, 52). However currently, there is a dearth of information on the effects of CPX on lung cancer. Ours is the first study to explore these effects and mechanisms.

ROS is an endogenous antioxidant that protects cells from oxidative stress (53). CPX not only increased ROS levels but the CPX inhibitory effects toward tumors were eliminated by using the ROS scavenger, NAC, which further confirmed that the ROS caused by CPX inhibited the proliferation of LUAD. Previously, CPX induced ROS by targeting DJ-1 (25), which is primarily oxidized to cysteine sulfonic acid which scavenges excessive ROS (53). We confirmed that ROS accumulation caused by CPX was associated with DJ-1 inhibition.

Previous studies reported that ROS is closely related to DNA damage and repair mechanisms (51). We showed that DNA DSBs were caused by CPX in a dose and time-dependent manner, and also activated the ATR-CHK1-P53 pathway which is an important mechanism during DDRs. Also, P53 is a downstream target of ATR and has a key role in controlling DNA damage-induced G1/S checkpoints (54). CPX significantly activated the ATR-CHK1-P53 pathway and arrested the cell cycle at the G1 phase. The expression of related G1 phase proteins was also decreased, including cyclin D_3_, CDK4, CDK6, and Rb. The ATR-CHK1-P53 pathway senses intrinsic damage and coordinates multiple pathways to arrest the cell cycle or induce apoptosis (55). We also observed that apoptosis was induced by CPX; previously, apoptosis was proposed as an effective therapeutic approach for tumor treatment (56). Thus, the DNA damage caused by CPX activated the ATR-CHK1-P53 pathway and caused cell cycle arrest, which ultimately led to apoptosis.

Furthermore, TOP2A was suppressed in the CPX treatment group. According to the TCGA database, TOP2A is one of the top 25 overexpressed genes in LUAD. Additionally, TOP2A expression levels are associated with a poor prognosis; previous studies reported that TOP2A could serve as a potential prognostic indicator and target for cancer therapy (42, 43). We not only confirmed that TOP2A knockdown inhibited LUAD, but it enhanced CPX anti-tumor effects. TOP2A is essential for DNA recombination, replication, transcription, and repair processes (33). After TOP2A is inhibited, LUAD processes cannot recover, DNA damage accumulates, generates more apoptosis, and further inhibits LUAD. Therefore, TOP2A has pivotal roles in CPX-induced LUAD inhibition, and suggests CPX exerts anti-LUAD activity, at least partially *via* TOP2A.

**Figure 8:**
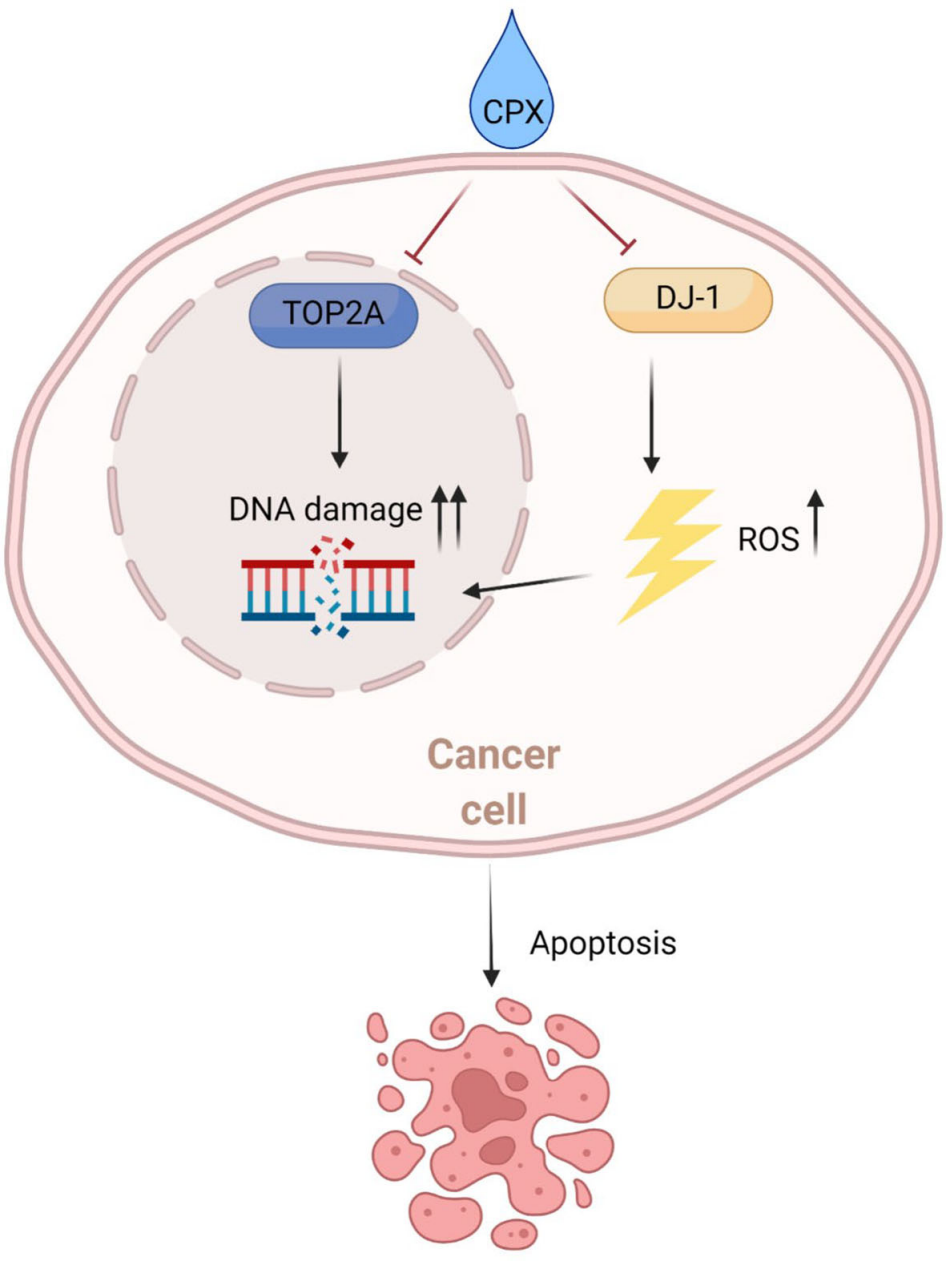
Schematic showing the potential therapeutic effects of CPX toward LUAD. Mechanistically, CPX causes DNA damage by inducing ROS and suppressing TOP2A expression, thereby blocking DNA repair, aggravating DNA damage, and causing apoptosis.

We hypothesize CPX induces ROS generation which leads to DNA damage. Not only that, CPX suppresses TOP2A which is crucial for DNA replication and repair, further aggravating DNA damage, and causing apoptosis (**Figure 8**).

While CPX inhibited LUAD cell proliferation by down-regulating TOP2A expression, the underlying mechanisms are unequivocally multifaceted. For example, CPX anticancer activities are dependent, in part, on its iron-chelating activity. Furthermore, CPX caused DNA damage which is related to iron chelation (30). Indeed, CPX reportedly inhibited ribonucleotide reductase which is essential for DNA synthesis, *via* iron chelation mechanisms (11). Whether TOP2A is related to CPX-mediated iron chelation is unknown, therefore further research is warranted.

Importantly*, in vivo* and *in vitro* CPX safety was verified. In previous studies, it was confirmed that the median lethal dose (LD50) value of CPX ranged from 1700 mg/kg to 3290 mg/kg in mice, rats, and rabbits (57, 58). Recently, CPX was shown to suppress tumor growth in myeloid leukemia, breast cancer, and colorectal tumor xenografts at 20–25 mg/kg/day, but displayed no toxicity to animals (25, 26, 59). Consistent with these observations, we showed that CPX inhibited LUAD tumor growth at 25 mg/kg/day and displayed no obvious toxicity. Additionally, a phase I clinical trial reported that CPX was safe and tolerable for leukemia patients (19); according to this clinical trial data, the peak serum concentration of CPX can reach more than 200 ng/ml after taking CPX. In terms of CPX applications for solid tumors, a multi-center phase I clinical trial in the USA is underway (NCT03348514). These data prove that CPX is an effective and safe potential anti-LUAD drug.

## Conclusions

Collectively and for the first time, we showed CPX had antitumor effects against LUAD. CPX also exerted a novel antineoplastic mechanism by suppressing TOP2A. Our *in vivo* studies showed that CPX was well tolerated in mice. CPX could function as a promising therapeutic agent and may provide significant benefits to patients with LUAD. Accordingly, more clinical trials and studies must be conducted to verify our data.

## Data Availability Statement

The original contributions presented in the study are included in the article/supplementary material. Further inquiries can be directed to the corresponding authors.

## Ethics Statement

The animal study was reviewed and approved by the Institutional Animal Care and Use Committee (IACUC), ZJCLA.

## Author Contributions

JY and GC wrote the manuscript, performed the experiments, and analyzed the data. KJ, ZZ, and LW participated in data collection and analysis. MX investigated the project. SY and JL were responsible for the research design. All authors contributed to the article and approved the submitted version.

## Funding

This study was supported by grants from the Chinese Medicine Research Program of Zhejiang Province (Grant No.2018ZZ014), Zhejiang Provincial Key Discipline of Traditional Chinese Medicine (Grant No.2017-XK-A32), the Fundamental Research Funds for the Central Universities (Grant No.2021FZZX005-32), the Natural Science Foundation of Zhejiang Province of China (Grant No.LSY19H160004), the Zhejiang Provincial Natural Science Foundation & Zhejiang Society for Mathematical Medicine (Grant No.LSY19H160002), the Zhejiang Provincial Natural Science Foundation (Grant No.LQ18C090005), and The Key Research and Development Project of Zhejiang Province (Grant No.2019C03071).

## Conflict of Interest

The authors declare this research was conducted in the absence of any commercial or financial relationships that could be construed as a potential conflict of interest.

## Publisher’s Note

All claims expressed in this article are solely those of the authors and do not necessarily represent those of their affiliated organizations, or those of the publisher, the editors and the reviewers. Any product that may be evaluated in this article, or claim that may be made by its manufacturer, is not guaranteed or endorsed by the publisher.
